# Oral Administration of Neratinib Maleate-Loaded Lipid–Polymer Hybrid Nanoparticles: Optimization, Physical Characterization, and In Vivo Evaluation

**DOI:** 10.3390/pharmaceutics17020221

**Published:** 2025-02-08

**Authors:** Radhika Rajiv Mahajan, Punna Rao Ravi, Sakshi Jadhav, Prinsi Kishorbhai Pansuriya, Bhushan Gopalsing Naik, Shalaka Hanmant Anture, Łukasz Szeleszczuk

**Affiliations:** 1 Department of Pharmacy, Birla Institute of Technology and Science Pilani, Hyderabad Campus, Jawahar Nagar, Kapra Mandal, Medchal District, Hyderabad 500078, Telangana, India; p20200469@hyderabad.bits-pilani.ac.in (R.R.M.); h20221460222@hyderabad.bits-pilani.ac.in (S.J.); h20221460210@hyderabad.bits-pilani.ac.in (P.K.P.); h20221460209@hyderabad.bits-pilani.ac.in (B.G.N.); h20221460202@hyderabad.bits-pilani.ac.in (S.H.A.); 2 Department of Organic and Physical Chemistry, Faculty of Pharmacy, Medical University of Warsaw, Banacha 1 Str., 02-093 Warsaw, Poland; lukasz.szeleszczuk@wum.edu.pl

**Keywords:** poor aqueous solubility, glyceryl distearate, hybrid nanoparticles, loading efficiency, solvent diffusion method, pharmacokinetic studies

## Abstract

**Background**: Neratinib maleate (NM), a tyrosine kinase inhibitor, is used in the treatment of breast cancer. Current oral therapy of NM suffers from low and variable bioavailability due to the solubility and permeability-related issues of the drug. To overcome the low oral bioavailability, the drug is recommended to be administered at high doses, causing severe gastrointestinal side effects leading to discontinuation of the drug therapy. **Methods**: In this work, NM-loaded lipid–polymer hybrid nanoparticles (NM-LPNs) were designed and optimized to improve the oral bioavailability of the drug. A systematic approach involving a screening design followed by an optimization design based on the principles of design of experiments (DoE) was used to prepare NM-LPNs. Minimum particle size (PS) ranging between 200 and 300 nm and maximum drug loading (DL (%)) were set as the target physicochemical properties. The optimized NM-LPNs, with a mean PS of 278.57 ± 21.16 nm and a DL (%) of 25.77 ± 1.11%, were further characterized for physicochemical properties, thermal and diffractometric analysis, stability, in vitro drug release, and oral pharmacokinetic studies. **Results**: The nanoparticles exhibited a burst release followed by a prolonged release up to 12 h in the in vitro drug release studies in pH 6.8 media. **Conclusions**: The mean C_max_ and the AUC_last_ values were found to increase significantly for NM-LPNs by 1.72 times (*p* < 0.01) and 1.58 times (*p* < 0.01), respectively, when compared to plain NM in the oral pharmacokinetic studies. The optimized NM-LPN formulation can reduce the oral dose of NM and, thereby, its dose-dependent side effects.

## 1. Introduction

Neratinib maleate (NM) belongs to the class of tyrosine kinase inhibitors used for breast cancer therapy. NM, being a BCS IV drug, similarly to the other APIs from this class, suffers from low and variable oral bioavailability due to low solubility, poor dissolution rate, and low permeability through the gastrointestinal membranes. NM is a salt (maleic acid) of a weakly basic drug (Neratinib). The solubility of NM decreases with an increase in pH. The drug is practically insoluble at pH > 6, slightly soluble in the pH range of 3 to 6, and soluble at pH < 3. Another reason for the low and variable oral bioavailability (11–39%) is the extensive gut-wall metabolism of the drug, specifically by the cytochrome P450 enzymes [1]. The available therapy of NM requires a high drug dose (290 mg/day) to overcome the low oral bioavailability. The current oral therapy (NERLYNX^®^ oral tablets) involves the administration of 290 mg of NM, divided into 6 tablets, which are taken together at one dosing point. The drug dose is divided into 6 tablets to make the dosing regimen amenable for dose titration (by reducing one tablet at a time) in the event of side effects in the patients. Therefore, the current oral therapy using the conventional tablets of NM suffers from patient compliance and convenience. In addition, the severe gastrointestinal side effects in some patients can lead to discontinuation of the therapy [2]. Designing novel drug delivery systems to improve the oral bioavailability of NM can reduce the dose and alleviate the dose-dependent side effects to improve patient compliance and effectiveness of the oral therapy of NM.

Nowadays, nanoparticulate systems are gaining much attention in solving problems related to dissolution and permeability. Reducing the particle size to a nano range helps improve bioavailability [3,4]. The primary reason is the enhancement of the surface area. Based on this primary reason, multiple probable secondary reasons are responsible for the increase in oral bioavailability, such as the following: (a) enhanced interaction between nanoparticles and gastric linings of mucous leading to increased movement of nanoparticles through the cellular membranes; (b) increased saturation solubility and increased dissolution rate; (c) decreased diffusion distance, causing increased concentration gradients in the respective dissolution environments; and (d) easy engulfment by macrophages and enterocytes [5,6]. All these advantages synergistically act and enhance the effectiveness of the nanoparticulate system to improve the therapeutic index of highly lipophilic molecules with aqueous solubility issues [7,8].

While developing nanoparticulate systems, especially for chronic therapies, the nature of excipients plays an important role. The excipients should be biodegradable and biocompatible so that the nanoparticulate systems are safe for long-term administration. Further, the excipients should also provide a good drug loading percentage (DL (%)), particularly for high-dose drugs. Drugs with low oral bioavailability usually need higher doses to deliver therapeutic efficacy. When high-dose drugs are formulated to be delivered as nanoparticulate systems, the overall mass of the dosage form depends on the DL (%). The lower the DL (%), the higher the mass of the nanoparticulate system required to be administered as one single dose. The relationship between DL (%) and the total mass of formulation to be administered are inversely related. Hence, while selecting the carriers for nanoparticulate systems, high solubility and/or DL (%) of the candidate drug molecule should be targeted.

Current research formulated a hybrid system, including glyceryl distearate (GDS) as a lipid and poly-lactic-co-glycolic acid (PLGA) as a polymer. Both the excipients are well-known USFDA-approved excipients from the class of lipids and polymers, respectively, for multiple routes of administration [9,10]. GDS consists of esters of palmitic (C16) and stearic (C18) acids, and the diester fraction is predominant. It has a melting point of 50–60 °C and an HLB value of 2. It is widely used as a lubricant for tablet and capsule manufacturing processes. It is also GRAS-listed and used in USFDA-approved marketed products for oral administration in humans. NM was found to have limited solubility in GDS. The solubility of the drug in the lipid carrier is vital for forming lipid nanoparticles with high drug DL (%). Therefore, a hybrid lipid–polymer nanoparticle system was designed by incorporating a polymer in addition to GDS in the formulation. PLGA has an established safety profile in humans, and most importantly, it is available in different grades with a range of lipophilic and hydrophilic properties. The lactic acid part of PLGA imparts hydrophobicity, whereas the glycolic acid part gives hydrophilicity to the polymer. The ratio of lactic acid to glycolic acid used in the synthesis of the PLGA determines the relative lipophilic or hydrophilic nature of the polymer [11]. In the current research work, PLGA 65:35 grade was used due to its relatively lipophilic properties compared to the other available grades of the polymer.

Different research groups explored a lipid–polymer hybrid system to improve the solubility and modify the release rates of several drugs for their effective oral delivery [12,13,14]. However, an extensive literature survey revealed that there are no published reports on hybrid lipid–polymer nanoparticulate systems for improving the oral bioavailability of NM. As per the available literature, Rahamathulla, M. et al. developed effervescent floating tablets of neratinib with a combination of polymers (Hydroxypropyl methylcellulose and Carbopol 940) to enhance the solubility and the gastrointestinal residence time of the drug in the upper GIT. The authors reported a prolonged floating time of > 12 h and sustained drug release of the drug for a period of 8 h in the in vitro drug release studies. However, in vivo pharmacokinetic studies were not reported by the authors [15]. Apart from that, in our earlier research works, we have developed and optimized a SEDDS and a mesoporous silica-based solid dispersion for NM. Both formulations were characterized for physicochemical properties, in vitro drug release, and in vivo pharmacokinetic studies. The optimized SEDDS and solid dispersion formulations improved the oral bioavailability of NM by 2.04 folds and 1.78 folds, respectively, compared to oral suspension of the drug [16,17]. Further, the combination of GDS and PLGA has not been explored to date for developing nanoparticulate systems to modify the release properties of hydrophobic drugs. Therefore, in this study, the novel hybrid lipid–polymer nanoparticulate system was designed, developed, and optimized using the principles of DoE. A mixed approach of screening followed by optimization was utilized in the study. The optimized NM-LPNs were lyophilized to evaluate for physicochemical characterization, in vitro drug release studies, stability studies, and oral pharmacokinetic studies.

## 2. Materials and Methods

### 2.1. Materials

The main analyte, Neratinib maleate (NM), and the internal standard, Rufinamide (only in the bioanalytical method), were provided as gift samples by MSN Laboratories Pvt. Ltd. and Dr. Reddy’s laboratories (Hyderabad, India), respectively. Lipids like Glyceryl monostearate, glyceryl distearate, Gelucire 59/14, Gelucire 44/14, Gelucire 48/16, Cetyl alcohol (Kolliwax CSA 50), Stearyl alcohol (Kolliwax SA), Myristyl alcohol (Kolliwax MA), Stearic acid 50 (Kolliwax S), palmitic acid, myristic acid, stearic acid, and tocopheryl polyethylene glycol succinate (TPGS) were gift samples from Gatte Fosse (Mumbai, India), BASF (Mumbai, India) and Merck (Mumbai, India), collectively. Poly-lactic-*co*-glycolic acid (PLGA) polymer of grade 65:35 (with acid termination and molecular weight of 15,000–40,000 Da) was purchased from Nomisma Healthcare Private Ltd. (Vadodara, India). Potassium chloride, Sodium chloride, etc., were acquired from SD Fine Chemicals Pvt. Ltd. (Mumbai, India). Acetonitrile (ACN) and methanol (MeOH) were purchased from Actylis (Bangalore, India), and the organic solvents were of the HPLC grade. Filtered MilliQ water was sourced from the Millipore (Millipore^®^, Burlington, MA, USA) unit established in the institute’s central analytical laboratory. Vab Biosciences (Hyderabad, India) provided the female Wistar rats for the pharmacokinetic evaluations.

### 2.2. Analytical and Bioanalytical Method

The in vitro samples were analyzed using high-performance liquid chromatography coupled with an ultraviolet (HPLC-UV) detector. The method was used to determine the DL (%), % entrapment efficiency (EE (%)), drug concentrations from in vitro drug release study samples, and assay of stability samples. The established HPLV-UV method’s partial validation was performed with parameters including linearity, accuracy, and precision. Using a Phenomenex Kinetex C18 column (5 µm particle size, 250 mm length and 4.6 mm internal diameter) in an isocratic mode, a mobile phase composed of 40% of MeOH (organic phase) and 60% of 0.1% *v*/*v* orthophosphoric acid in the water at a fixed pH value of pH 2.5 ± 0.1 (aqueous phase) was pumped into the column. A flow rate of 1 mL/min was used with the detection wavelength of 268 nm, and the column was kept at a constant temperature of 25 °C. An amount of 10 µL of the sample was injected for each sample analyzed. The calibration range of the method was between 1000 and 15,000 ng/mL, with 46 ng/mL as the limit of quantification. Our research group developed and validated a bioanalytical method for the quantification of NM using an HPLC attached with a UV detector. The details are discussed in the published article [18].

### 2.3. Selection of the Formulation Components

#### 2.3.1. Screening of Lipid and Organic Solvent

An accurately weighed quantity of 5 mg of the drug was added to 750 mg of different solid lipids selected for the screening studies. The weighed mass was transferred to an HPLC clear glass vial. All these vials were then placed in a water bath maintained at a temperature of 80 °C. At different time intervals, the vials were vortexed and observed for the solubilization of NM in the respective lipids. The lipids were screened based on the visual observation and a quantitative analysis to evaluate the solubility of NM in the lipids.

The organic solvent was selected based on the drug solubility and its capacity to solubilize other organic phase components. An equal amount of drug (5 mg) was added to 1 mL volume of various organic solvents, including MeOH, dimethyl sulfoxide (DMSO), ACN, ethyl acetate, chloroform, and dichloromethane. These solution tubes were visually examined for the solubility of the drug, followed by extra additions of the drug upon complete solubilization of the previously added drug. The organic solvent with a clear homogenous appearance was finalized.

#### 2.3.2. Stabilizer Screening Studies

An amount of 5 mg of NM was added into 2% *w*/*v* (20 mg/mL) and 5% *w*/*v* (50 mg/mL) solutions of different solubilizers, including TPGS, Poloxamer 407, Gelucire 44/14, Gelucire 48/16, Gelucire 59/14, HPMC E5 LV, PVP K30, Tween 80, Tween 20, and PVA in filtered MilliQ water. All the solutions were vortexed and mixed on a rotating device, ROTOSPIN ™ (Tarsons, Kolkata, India), for 12 h. After 12 h, the solutions were centrifuged at a speed of 11,490× *g* for 15 min, followed by the collection of the supernatant. The supernatant was diluted 10× with a suitable diluent, and the drug content in these stabilizer solutions was determined using the HPLC method described in Section 2.2.

### 2.4. Method of Preparation for NM-LPNs

The organic phase was prepared by dissolving 20 mg of NM, 28.5 mg of PLGA (65:35), and 4 mg of GDS (Precirol^®^ ATO 5) in 1.5 mL of DMSO. The organic phase was heated in a water bath at a temperature of 65 °C (5 °C higher than the melting point of the GDS, i.e., 55–60 °C) to dissolve the lipid. The aqueous phase was prepared by dissolving Poloxamer 407 (300 mg) in chilled filtered MilliQ water (15 mL) to yield a 2% *w*/*v* solution of the stabilizer. The pH of the aqueous phase was adjusted to a pH of 6.65 ± 0.5 using 50 mM ammonium acetate solution. The aqueous phase was heated to 53 ± 2 °C just before the addition of the organic phase. A pre-emulsion was formed by slowly adding the organic phase into the aqueous phase with continuous stirring at 300 rpm on a magnetic stirrer. The organic phase was added at a rate of 1 mL/min into the aqueous phase using a 1 mL syringe attached to a 25-gauge needle. Following the complete addition of the organic phase into the aqueous phase, the pre-emulsion formed was subjected to a homogenization process.

Kinematica PT 3100 D homogenizer attached with a 12 mm diameter probe was used to homogenize the pre-emulsion for 2 min at a speed of 7000 rpm. The resultant dispersion was then subjected to mixing on a magnetic stirrer at 200 rpm at 25 ± 2 °C (controlled room temperature conditions). Finally, the dispersion was diluted by adding 15 mL cold-filtered MilliQ (maintained at 10 ± 0.5 °C) at a rate of (2 mL/min) to the 15 mL nanoparticle formulation preparation. The formulations were then stirred until the surface foam was dispelled from the nanodispersion (approximately 20–25 min), labeled, and stored in refrigerated conditions until further use.

### 2.5. Application of DoE to Design, Develop, and Optimize NM-LPNs

During the preliminary formulation trials, it was observed that multiple formulation-related factors and manufacturing process-related factors affected the critical responses (desired physicochemical properties of NM-LPNs). Therefore, a screening design was used to find the vital few out of the many trivial factors affecting the responses of the NM-LPNs. The screening design was performed by adding a few center point runs to check if the relationship between the factors and the responses was linear or non-linear. Based on the results, which suggest a non-linear relationship, an RSM was used to optimize the relationship between the critical factors and the responses. The RSM design helped determine the final composition and the manufacturing process conditions to prepare NM-LPNs with the maximum desirability value. This systematic DoE approach avoids the use of excess resources and simplifies the process of arriving at the final optimum solution to prepare NM-LPNs. Design Expert^®^ (version 10.0.3) software by Stat-Ease Inc. (Minneapolis, MN, USA) was utilized to build the screening and optimization design matrices, perform regression analysis of data obtained from DoE trials, and finally apply the desirability function to arrive at the optimum solution.

### 2.6. The Selection of the Screening Design

A fractional factorial design (2^6−2^ Resolution IV) was used for the screening of 6 factors identified from the preliminary formulation trials. Table 1 enlists the 6 factors with the respective higher (+1) and lower (−1) levels (in their respective original scale) selected for the design. In the screening design, each factor was studied at two levels, including three center points, resulting in 19 runs. Center points were included to check the presence of curvature in the relationship between the factors and the responses. The PS, ZP, PDI, and DL (%) of the NM-LPNs were selected as the responses in the screening design.

#### 2.6.1. Response Surface Methodology for Optimization Using Box–Behnken Design (BBD)

Out of 6 factors analyzed using screening design, polymer amount, lipid amount, and Tween 80 concentration in the organic phase were identified as the statistically significant factors that were taken up for optimization using RSM. Out of the four responses studied in the screening design, only two responses, namely PS and DL (%), were considered in the optimization design. The three remaining factors that were found to be statistically insignificant in the screening design were fixed at specific values in the optimization design. The temperature of the dilution phase was set at 10 °C, the homogenization time was set at 2 min, and the homogenization speed was fixed at 7000 rpm in all the optimization trials. BBD, a type of RSM design, was employed to relate the critical factors (X_1_, X_2_, and X_3_) to the response variables (Y_1_ and Y_2_) by a mathematical equation. A total of 15 experimental runs, including 12 BBD runs and 3 center point runs, were involved in the design matrix. Randomization in the order of the experiments was performed to avoid bias, and all the runs were performed in one block. The critical factors and their levels are mentioned in Table 2.

#### 2.6.2. Solution Identification Using Desirability Function and Validation of RSM Design

The present research aimed to develop NM-LPNs with small PS and high DL (%). The objective function for each response (i.e., PS (Y_1_) and (Y_2_)) was defined prior to the experimental runs. A desirability function-based simultaneous optimization technique was used to optimize the two responses simultaneously. A solution(s) with an overall desirability of ‘1’ is regarded as ideal. The solution provided by the software with the highest desirability value was finalized as the optimized conditions for the preparation of NM-LPNs.

To verify the RSM design, six replicate formulations of the optimized NM-LPNs were manufactured based on the conditions proposed by the desirability function’s solution. The formulations were evaluated for their PS and DL (%). A statistical hypothesis testing, based on interval estimation, was used to analyze if the expected PS and DL (%) values (calculated by substituting the levels of factors suggested by the solution in the regression equation for PS and DL (%)) were similar or different from the observed response values. The software determines the 95% confidence interval (CI) for PS and DL (%) particular to the selected solution (with maximum desirability function). The regression equations for both responses are to be considered valid and reliable only if the observed mean values obtained from the six replications of the formulation lie within the minimum and maximum values set by the software (with 95% CI).

### 2.7. Characterization of NM-LPNs

#### 2.7.1. Particle Size (PS), Polydispersity Index (PDI), and Zeta Potential (ZP)

Zetasizer nano ZS (Malvern Instruments, Worcestershire, UK) was used to determine the PS, PDI, and ZP of NM-LPNs. The analysis was based on the principle of dynamic light scattering. A fixed backscatter angle 173^0^ with a 633 nm laser was used to perform all the measurements. Each sample was diluted (200×) with filtered MilliQ water and placed in the sample compartment to equilibrate at 25 °C for 180 sec. The same diluted sample was used to determine the ZP. The cuvettes used for ZP are different and have conducting plates on both sides. The values of PS, PDI, and ZP of each sample were reported as the average of triplicate measurements.

#### 2.7.2. Entrapment Efficiency (EE (%)) and Drug Loading (DL (%))

For the freshly prepared nanoparticle batches, as per screening and optimization design, an indirect method was used to determine the EE (%) and DL (%), which only considered the unentrapped drug for the final calculation. Freshly prepared NM-LPN nanosuspension was centrifuged at 4402× *g* for 15 min to separate the pellets of NM-LPNs from the supernatant. Then, the supernatant was collected into a separate tube and centrifuged at a higher speed of 11,269× *g* for 30 min to facilitate the sedimentation of nanoparticles and obtain a clear supernatant. A sample was collected from the supernatant and analyzed to determine the amount of unentrapped NM present in it. The EE (%) and DL (%) of each formulation were calculated using the formula given in Equation (1) and Equation (2), respectively [19].

(1) $$EE\left(\%\right)=\frac{W_{Added}-W_{Supernatant}}{W_{Added}}\times 100$$

(2) $$DL(\%)=\frac{W_{Added}-W_{Supernatant}}{W_{NM+Excipients}}\times 100$$

where W_Added_ is the initial amount of NM added in the organic phase during the preparation of NM-LPNs; W_Supernatant_ is the amount of NM present in the clear supernatant obtained after the second centrifugation process described above; $W_{NM+Excipients}$ is the total mass of the formulation calculated by adding the weight of the drug and all the excipients added in the formulation.

It was difficult and tedious to determine the DL (%) by the direct method for each formulation prepared during screening and optimization studies. Hence, the DL (%) by direct method was determined only for the lyophilized nanosuspension formulation (5% mannitol as cryoprotectant) of optimized NM-LPNs. The lyophilized NM-LPNs were weighed at a fixed mass and dissolved in a mixture of organic and aqueous solvents (0.5 mL filtered MilliQ + 0.5 mL 0.1 N HCl + 1 mL of DMSO) to extract NM. The extraction solution was then diluted appropriately and used to quantify NM using the HPLC method. DL (%) was reported as the weight of NM in the total weight of the formulation, including the drug and excipients. The formula used is mentioned in Equation (3) [19].

(3) $$DL\left(\%\right)=\frac{W_{NM\;in\;Lyophillized\;formulation}}{{W_{NM}+W}_{Total\;weight\;of\;excipients}}\times 100$$

where W_NM in Lyophilized formulation_ is the amount of NM obtained after extraction of the drug from the fixed mass of Lyophilized formulation, W_NM_ is the initial amount of NM added in the organic phase, and W_Total weight of excipients_ is the addition of weight of all the excipients used while preparing the formulation of NM-LPNs.

#### 2.7.3. Differential Scanning Calorimetry (DSC)

Plain NM, PLGA, GDS, physical mixture of NM, PLGA, and GDS, and lyophilized NM-LPNs were analyzed using DSC-60 (TA-60 WS, Shimadzu, Kyoto, Japan) for their calorimetric properties. The weighed mass of samples was poured into the aluminum pans, followed by crimping with a small flat aluminum lid. The equilibration of the loaded samples was performed at 30 °C before the start of the run. The temperature range for the analysis was set between 30 and 250 °C at a uniform heating rate of 10 °C/min. The inert atmosphere was maintained in the sample loading chamber by purging nitrogen at a flow of 50 mL/min.

#### 2.7.4. Powder X-Ray Diffraction (pXRD) Analysis

The pXRD diffractograms of plain NM, GDS, PLGA (65:35), physical mixture of plain NM, GDS, and PLGA, blank LPNs, lyophilized NM-LPNs, and mannitol were recorded using a powder X-ray diffractometer (Rigaku Ultima IV, Tokyo, Japan) with a copper anode (1.54 Å) at a 60 mA current and voltage of 60 kV. All samples were analyzed in the scanning range of 5–40 degrees at a rate of 2 degrees/min, with a step width of 0.01 degrees, using a scintillation counter detector. The values for each X-ray diffractogram were collected and analyzed using Origin software (10.1.0.170 version). The 2θ values were plotted on the *x*-axis, and the intensity on the y-axis.

#### 2.7.5. Scanning Electron Microscopy (SEM)

The surface morphology of the optimized NM-LPNs was examined under a scanning electron microscope (FESEM, FEI, Apreo LoVac, Thermo Fisher Scientific, Waltham, MA, USA). A 1:10 dilution of the lyophilized NM-LPNs was prepared and centrifuged at a speed of 11,490× *g* for 15 min. After the centrifugation, the supernatant was discarded, and the pellet formed was dispersed in a small volume of 100 μL. Furthermore, 10 μL of the concentrated NM-LPNs suspension was placed on a clear glass coverslip and dried overnight at room temperature. The coverslips were placed on the appropriate sizes of the carbon tapes pasted on the carbon stubs and were then sputter-coated with gold with a thickness of 2–5 nm using a sputter module (Leica EM ACE200, Wetzlar, Germany). Argon gas was used to maintain the inert atmosphere in the chamber. Multiple images of these gold-layered samples were captured at an acceleration voltage of 10 kV. The images were analyzed at different magnification scales as per the requirements.

### 2.8. Stability Studies

Samples of the optimized NM-LPNs were kept in a stability chamber (Remi, Mumbai, India) for three months, in line with the ICH guidelines, at two different conditions: CRT of 25 ± 2 °C with RH of 60 ± 5% and an accelerated condition of 40 ± 2 °C with RH of 75 ± 5%. Airtight centrifuge tubes containing the samples (~100 mg of lyophilized powder of NM-LPNs) were sealed and loaded into the respective chambers. Samples were collected every month for three consecutive months. At each time point, the assay and the physical properties of the samples were evaluated. The data from the monthly sample collections were compared with the freshly prepared formulation at the initiation of the stability study. The percentage deviation of the assay was calculated with reference to the initial assay (100%). Each stability sample was tested in triplicates.

### 2.9. In Vitro Drug Release of Plain NM and NM-LPNs

In vitro release studies were performed using a surfactant solution of 2% *w*/*v* Gelucire 44/14 in pH 6.8 and 7.4 to mimic the lower GIT and systemic conditions, respectively. A quantity of 40 mL of dissolution media was poured into an amber-colored 50 mL falcon tube. The dissolution media was heated to a physiological temperature of 37 °C before any addition, and zero samples were collected. An accurately weighed mass of 36 ± 0.5 mg of the lyophilized formulation (equivalent to 0.5 mg of NM) was added into the preheated dissolution media. The exact amount of drug was used as a control for the dissolution study. Samples of 1 mL volume were withdrawn at specific intervals of 0, 0.25, 0.50, and 1 h in the case of plain NM. The time points selected for the NM-LPNs included 0, 0.25, 0.75, 1, 3, 5, 7, 9, 12, 20, and 28 h. Each sample was centrifuged at 11,490× *g* for 15 min. The supernatant was separated into a fresh tube, and the sedimentation was reconstituted with 1 mL volume of fresh buffer, vortexed, and mixed again in the respective sample mixtures. The dissolution data fit into different release kinetics models. The best-fit model was selected based on the maximum R^2^ value [20].

### 2.10. In Vivo Oral Pharmacokinetic Studies

In vivo oral pharmacokinetic studies were conducted in female Wistar rats (*n* = 3) weighing approximately 250 g to determine the oral pharmacokinetics of the optimized NM-LPNs. The Institutional Animal Ethics Committee of BITS Pilani Hyderabad Campus, Hyderabad, India, examined and approved the protocol for all the in vivo studies (Protocol No.: BITS-HYD-IAEC-2023-18). The rats were acclimated (temperature of 22 ± 1 °C; RH of 55 ± 10% and 12 h light–dark cycle) for a week before the dosing of treatments in the institute’s animal facility (Animal house registration number 1912/PO/RE/S/16/CPCSEA). The rats were kept for a 12 h fasting period before the study was initiated, with water availability as ad libitum. Aqueous suspension of lyophilized NM-LPNs (containing 0.25% *w*/*v* of NM in the nanosuspension) was prepared by dispersing the accurately weighed amount of ~95 mg (calculated based on the DL (%) of the NM-LPNs) of lyophilized NM-LPNs in 1 mL of MilliQ water. The formulation was administered at a dose of 10 mg/kg in the animals using a rat oral gavage. Free water access was removed to the animals following the administration of the treatments. Water was administered manually at specific intervals. The protocol for water administration was as follows: 0.5 mL at the first time point after dosing and 0.25 mL at every time point up to 8 h. The centrifuge tubes with 1.5 mL capacity were prepared for blood collection by adding 25 μL of anticoagulant solution (4.5% *w*/*v* EDTA solution; one-part EDTA solution to nine parts blood). Approximately 200 μL of blood was collected at each sampling point to obtain approximately 100 μL of plasma. The blood samples were withdrawn at pre-dose and 1, 2, 3, 4, 6, 8, 12, 15, 22, and 30 h following the administration of the treatment. Free access to food and water was provided to animals after the 8th hour of administering the treatments. The plasma concentration data for plain NM, presented in our previously published work, were used to compare the pharmacokinetic performance of the formulations [17]. All the collected samples were analyzed by using a validated HPLC-UV method published earlier [18]. The calibration equation was used to determine the unknown concentrations, and the data were analyzed using Phoenix^®^ WinNonlin software (version 8.3.5.340, Certara Inc., Durham, NC, USA). Noncompartmental analysis (NCA) was found suitable for determining the pharmacokinetic parameters from the plasma time course data obtained from the oral administration of NM-LPNs.

## 3. Results and Discussion

### 3.1. Preliminary Formulation Trials

The NM-LPNs are a combination of a lipid and a polymer. Initially, the solubility of NM was checked in different lipids with the procedure mentioned in Section 2.3.1. Based on the results, GDS had shown the maximum solubility of NM (15 mg/750 mg of lipid). In contrast, to translate the formulation to the human dose of NM, i.e., 240 mg, it would be unrealistic only to use this lipid. Hence, it was decided that in addition to the selected lipid, a biocompatible polymer should be used as a hybrid combination. The very first choice of polymer was PLGA due to the availability of different grades of the polymer. NM has more hydrophobicity; hence, PLGA polymer grades with higher polylactic acid content than polyglycolic acid content were selected. Later, based on the requirements of releasing the drug from the formulation or considering once-a-day dosing frequency of NM, 65:35 was the grade of choice. Hence, PLGA (65:35) and GDS were finalized as the polymer and lipid for the formulation of NM-LPNs.

Dissolving a maximum amount of the drug was the most important criterion for choosing the organic solvent. Unfortunately, NM was found to be insoluble in most of the organic solvents, including chloroform, ethyl acetate, acetone, N-methyl pyrrolidone, etc., except methanol and DMSO. In addition to NM, the selected organic solvent must dissolve PLGA and the lipid. Hence, to solubilize all the components, including NM, lipid, and polymer, DMSO was selected over methanol as the organic solvent due to its greater capacity to dissolve NM.

**Figure 1:**
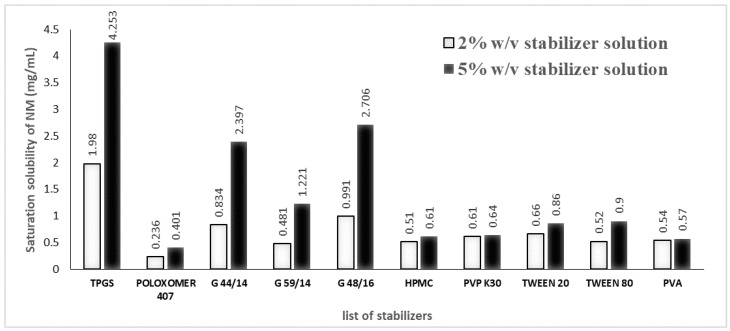
Saturation solubility of NM in aqueous solutions (2% *w*/*v* and 5% *w*/*v*) of different stabilizers.

A suitable stabilizer was selected after performing a drug-stabilizer solubility study, as mentioned in Section 2.3.2. Amongst all the stabilizer solutions of different percentages, whichever stabilizer solution that showed the least amount of NM solubilization was given priority over the remaining. To explain it further, as there is the least amount of NM solubility in the aqueous medium, the chances of its encapsulation in the lipid–polymer hybrid system increase significantly. Figure 1 shows the solubility data of NM in different stabilizer solutions. Based on the results, Poloxamer 407 at 2% *w*/*v* concentration was finalized. Hence, from the preliminary data, GDS, PLGA (65:35), DMSO, and 2% Poloxamer solution were finalized as the lipid, polymer, organic solvent, and stabilizer solution, respectively, in the formulation.

### 3.2. The Manufacturing Process for NM-LPNs

**Figure 2:**
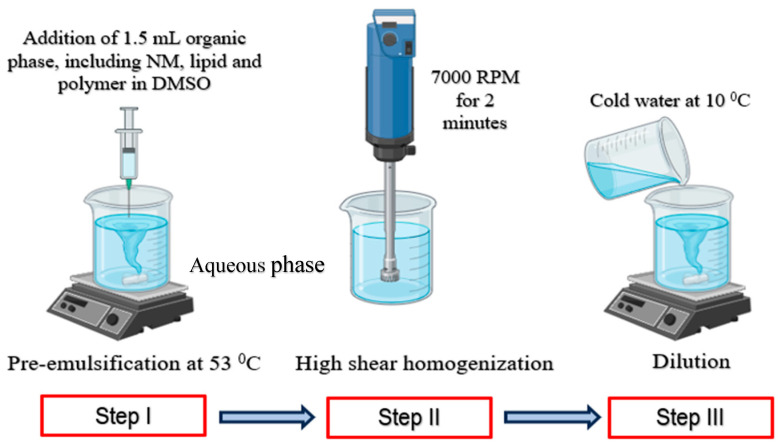
Stepwise process for NM-LPN formulation.

After a series of trials using different manufacturing processes, a three-step process was finalized, including pre-emulsification with heating using a magnetic stirrer, followed by high-shear homogenization and dilution with cold water as the third and last step. The diagrammatic representation of the process is shown in Figure 2.

### 3.3. Evaluation of Screening and Optimization Design to Obtain the Optimized NM-LPNs

Deciding the working ranges for the formulation components as well as the process parameters is one of the crucial steps of formulation development using the principles of DoE. The material attributes involved were polymer amount, lipid amount, and Tween 80 concentration in the organic phase, and the process parameters were temperature of the dilution phase, homogenization speed, and homogenization time. As there were more than three factors affecting the final product quality, we decided to go with a hybrid design, which includes both screening and optimization. The screening design helps to identify the vital few from the trivial many. Before optimization, the factors that did not impact the critical quality attributes of the product were excluded.

#### 3.3.1. Screening Design to Evaluate the Vital Few

A fractional factorial design was employed to identify critical factors from the six overall factors. The six factors were a mixture of material attributes (such as concentrations of polymer, lipid, surfactant, etc.) and process parameters (such as homogenization time and speed, etc.). A Resolution-IV fractional factorial design (2^6−2^ Resolution IV) with a total of 19 runs, including 3 center points, was found to be most suitable for screening significant factors (Table 1) used to identify the critical factors from the 6 factors. In this design, the main factors are not aliased or confounded with other main effects and 2-level interaction terms. Therefore, it is an appropriate screening design to identify the critical main effects. Additionally, the three center point runs assist in determining if there was any curvature in the relationship between the main effects and the responses.

The PS, PDI, ZP, and DL (%) were observed during the screening design as the responses. Plots such as Pareto charts and half-normal probability plots were employed as diagnostic tools to determine the statistically significant factors influencing the responses. This was further verified based on the F-values derived from the ANOVA of the regression models for each response. It was discovered that regression models for three responses namely, PS, PDI and DL (%) were significant (*p* < 0.01). Curvature was only found to be important in the DL (%) regression model (*p* < 0.01).

Statistical analysis was performed using the appropriate statistical tools of ANOVA for the regression models of the three responses, viz., PS, PDI, and DL (%). In the regression model for PS, lipid amount and Tween 80 concentration in the organic phase were found to be the statistically significant factors. In the case of PDI, the effect of polymer amount and lipid amount were found to be significant. Meanwhile, for DL (%), only the lipid amount was found to be significant, considering the *p*-values with a 95% CI and combining all factors affecting the effects in DL (%). Based on the results obtained in the screening design, out of the 6 factors, polymer amount, lipid amount, and Tween 80 concentration in the organic phase were finalized as critical factors. Out of the three responses, since PDI showed no specific variation and a similar trend to that of PS across the screening experimental runs, only PS and DL (%) were considered responses in the optimization studies.

#### 3.3.2. Response Surface Methodology-Based Optimization Design

Three factors—Tween 80 concentration in the organic phase (X_1_), lipid amount (X_2_), and polymer amount (X_3_)—were found to significantly affect the critical responses PS and DL (%). A Box–Behnken Design (BBD) was selected to study the effect of the three screened factors on the critical responses of the NM-LPNs. BBD was selected over central composite design (CCD) because those experimental runs where the levels of all the factors are at their low level and high level (−1, −1, −1 and +1, +1, +1, respectively) are avoided. In addition to this reason, fewer runs are involved as a part of BBD when compared to CCD. Hence, BBD was used as a response surface methodology to relate the independent variables or the critical factors (X_1_, X_2,_ and X_3_) to the response variables (Y_1_ and Y_2_) by a mathematical equation. Design Expert^®^ (version 13.0.15, Stat-Ease, Minneapolis, MN, USA) was used to build the design matrix. A total of 15 experimental runs, including three center point runs, were involved in the design matrix. The results are mentioned in Table 3.

PS ranged from 168 to 364.3 nm, while DL (%) ranged from 3.10 to 26.7% in the set of 15 experimental runs. The data obtained from executing the experimental runs as per the design matrix were exposed to statistical evaluations to determine the best-fit model. For DL (%), the best-fit model was found to be a quadratic model, while a 2FI model was found to be a better fit for PS. After checking the *p*-values from the ANOVA table, the statistically insignificant terms having a *p*-value > 0.05 were dropped. Residuals were randomly distributed across the zero line for all the regression models, with no specific trend. The revised ANOVA results from the regression analysis of the data for each of the two response variables adjusted for significant terms are given in Table 4.

#### 3.3.3. Effects of Critical Factors on the Responses

##### Particle Size (PS)

PS was decided to be one of the critical responses, as the fate of particles’ absorption in vivo depends on their size. The nano size range expected to have better uptake is from 200 to 300 nm. Hence, we also decided to target the same range for PS. The best-fit model for PS was found to be the reduced version of the 2FI model (*p* < 0.01) with a transformation of Log_10_, and the lack of fit was found to be insignificant (*p* > 0.05). Equation (4) elaborates on the relationship between PS and the critical factors.

(4) $$PS=2.39-0.01X_{1}-0.04X_{2}+0.07X_{3}+0.04X_{1}X_{2}+0.05X_{2}X_{3}$$

**Figure 3:**
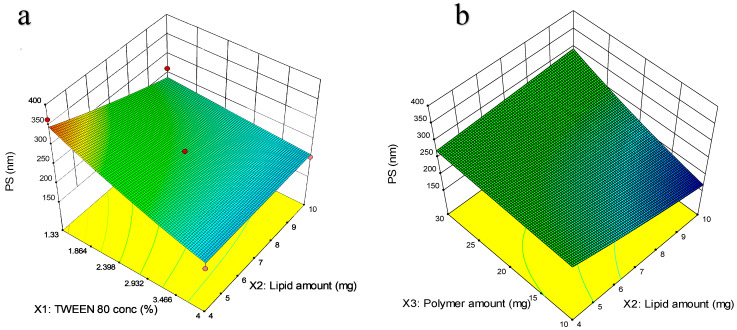
Illustrations of the impact of significant factors on the PS of the optimized NM-LNPs using 3D response surface plots: (**a**) X_1_X_2_ vs. PS; (**b**) X_2_X_3_ vs. PS.

In Figure 3a, the 3D graph showed that at a fixed level of polymer amount of 20 mg, an increase in Tween 80 concentration in the organic phase resulted in a significant decrease in particle size from 340 nm to 180 nm. An increase in surfactant concentration is supposed to reduce the interfacial tension and create steric hindrance on the LPN surface to prevent the accumulation of individual particles and improve stability. On the other hand, it was observed that increasing the lipid amount slightly increased the PS from 180 nm to 220 nm.

In Figure 3b, the 3D graph showed that at a fixed level of Tween 80 concentration of 2.66%, an increase in lipid amount and polymer amount from 4 mg to 10 mg and 10 mg to 30 mg, respectively, resulted in increased PS. The reason could be the increased viscosity of the organic solution due to higher concentrations of solids in it. The viscosity directly affects the efficiency of size reduction by homogenization as more force is required to break the particles efficiently.

##### Drug Loading (DL (%))

DL (%) was decided to be the second critical response, as it forms the basis for determining the mass of the optimized formulation needed to administer the required drug dose. The higher the DL (%) of the NM-LPNs, the lesser the overall mass of formulation required to administer the required dose of NM. Hence, maximum DL (%) was set as the target. The best-fit model for DL (%) was found to be the reduced version of the quadratic model (*p* < 0.01) without any transformation, and the lack-of-fit was found to be insignificant (*p* > 0.05).

(5) $$DL\left(\%\right)=22.50-2.31X_{1}-3.53X_{2}-1.32X_{3}+6.00X_{1}X_{3}-6.01X_{2}X_{3}-7.45X_{1}^{2}-4.57X_{3}^{2}$$

**Figure 4:**
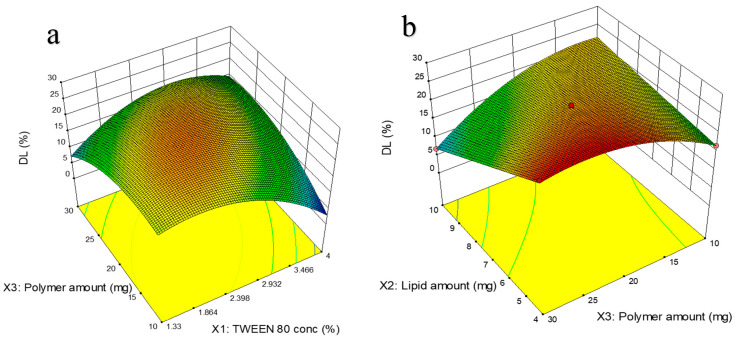
Illustrations of the impact of significant factors on the DL (%) of the optimized NM-LNPs using 3D response surface plots: (**a**) X_1_X_3_ vs. DL (%); (**b**) X_2_X_3_ vs. DL (%).

Figure 4a showed that at a fixed amount of lipid (7.5 mg), the increase in surfactant concentration from 1.33% to 2.4% resulted in an increase in DL (%) from 14% to 19%. At the same time, the increase in surfactant concentration from 2.4% to 4% showed a decrease in DL (%) from 19% to 5%. This suggests that an optimum level of surfactant is ideal to achieve higher DL (%). At higher levels of the surfactant, the solubility of the drug in the continuous phase increases, and the drug partitions more into it, thereby reducing the DL (%). An increase in polymer amount slightly increased the DL (%), particularly when the surfactant concentration was in the higher range (2.4% to 4%). In general, the amount of drug loaded in the nanoparticles increases with the amount of carrier due to the availability of more polymer molecules to interact in the internal phase, thereby resulting in higher DL (%).

In Figure 4b, the 3D response surface plot showed that at a fixed value of Tween 80 concentration of 2.6%, at lower amounts of polymer (in the range of 10 to 20 mg), increasing the lipid amount increased the DL (%). However, at higher amounts of polymer (in the range of 20 to 30 mg), increasing the lipid amount decreased the DL (%). An increase in polymer did not have a significant effect on DL (%), particularly when the polymer amount was low (in the range of 4 to 7 mg). However, the DL (%) decreased with an increase in polymer amount when the lipid amount was high (in the range of 7 to 10 mg). Overall, it can be inferred that a higher polymer amount and lower lipid amount had a positive impact on drug loading, which could be due to the slightly better affinity of the drug to the polymer than the lipid.

### 3.4. Determination of the Final Optimized Conditions

In the BBD-derived experimental space for three critical factors, it was found that DL (%) varied from 3.01 to 26.70%, and PS varied from 168 to 364.3 nm. Since three critical factors are involved, it becomes crucial to identify the optimal conditions such that the desired PS and DL (%) can be obtained. A simultaneous optimization technique was used to find the optimal conditions of the factors. The numerical optimization technique was used to set the goals for responses. For the DL (%), the goal was set as ‘maximize’, and the lower and upper limits were selected by the Design Expert software based on the experimental data. The goal set for PS was ‘is in range’. After setting these goals, several solutions containing different compositions of the factors X_1_, X_2,_ and X_3_ were provided. The proposed solution (levels of the factors) for the optimized NM-LPNs were as follows: 3.07% Tween 80 concentration in the organic phase, 4.00 mg lipid amount, and 28.42 mg polymer amount for the factors X_1_, X_2,_ and X_3_, respectively. The formulation prepared by this solution is predicted to have a DL of 26.88% and 255.67 nm. This solution is selected as it has the highest desirability (desirability = 1). For validation, six replicate batches with the composition given by the desirability function of the optimized NM-LPNs were prepared. Wilcoxon signed-rank test at α = 0.05 was used to compare the observed and predicted values statistically. The 95% CI for PS and DL (%) was 218.86–296.63 nm with a standard deviation of 21.16 and 24.68–29.09% with a standard deviation of 1.11, respectively. The mean value obtained from the validation for PS was 278.57 ± 21.16 nm and for DL (%) 25.77 ± 1.11%. Both the values were well within the established limits; hence, the validation was claimed as successful, and this solution was finalized as the optimized solution. Further, the optimized NM-LPNs were lyophilized to evaluate for physicochemical characterization, stability studies, in vitro drug release studies, and in vivo pharmacokinetic studies.

### 3.5. Physicochemical Characterization of NM-LPNs

#### 3.5.1. Differential Scanning Calorimetry

**Figure 5:**
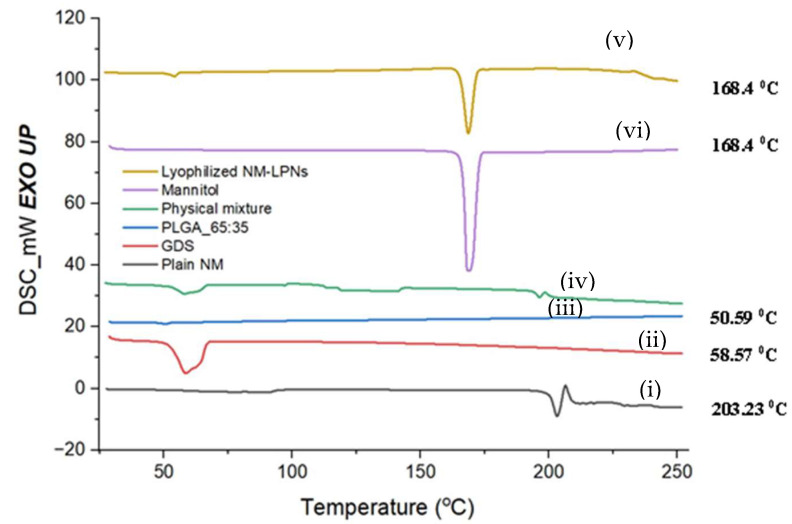
DSC thermograms of (i) plain NM, (ii) GDS, (iii) PLGA_65:35, (iv) physical mixture of plain NM, GDS, and PLGA, (v) lyophilized NM-LPNs, and (vi) mannitol.

The DSC thermogram for plain NM showed a sharp endothermic peak at 203 °C (Figure 5). GDS showed a broad endothermic peak in the range of 60–65 °C, which corresponds to the reported melting point of the lipid [21]. The thermogram of PLGA (65:35) showed a glass transition at 50 °C. However, there was no sharp melting endothermic peak for PLGA (65:35), indicating the amorphous nature of the polymer. A high-intensity peak was obtained for lyophilized NM-LPNs due to mannitol (used as a cryoprotectant in the lyophilization of NM-LPNs), which correlated with the melting endothermic peak observed in the thermogram of pure mannitol. Apart from the endothermic peak of mannitol, no other endothermic peak was observed in the DSC thermogram of lyophilized NM-LPNs. This suggests the successful loading of NM either in molecular or amorphous form in nanoparticles (NM-LPNs).

#### 3.5.2. Powder X-Ray Crystallography

**Figure 6:**
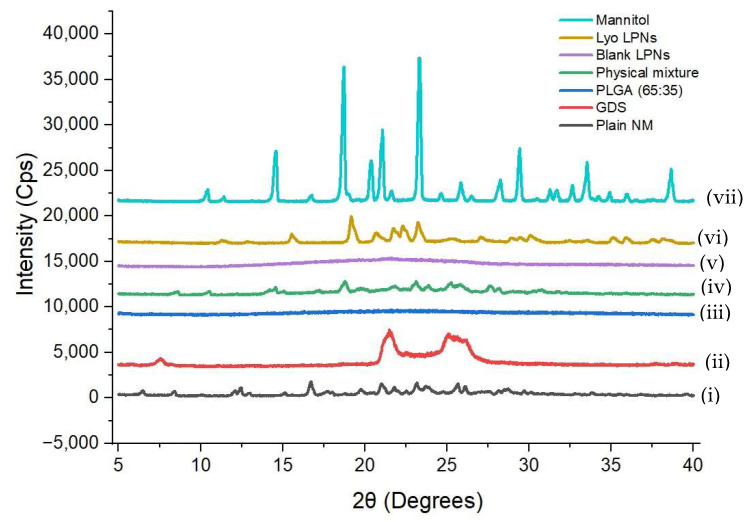
The pXRD graphs of (i) plain NM, (ii) GDS, (iii) PLGA (65:35), (iv) physical mixture of plain NM, GDS, and PLGA, (v) blank LPNs, (vi) lyophilized NM-LPNs (vii) mannitol.

The pXRD diffractograms of various samples analyzed in the study are presented in Figure 6. Plain NM showed sharp intensity peaks at 2θ values of 6.48, 8.42, 12.10, 12.42, 13.00, 16.72, 19.74, 21.80, 23.18, 23.72, 25.68, 26.14, 28.74, and 29.74 degrees indicating the crystalline nature of the drug powder. The pXRD analysis of GDS showed a semi-crystalline property of the lipid. In contrast, the PLGA appeared to be completely amorphous, which correlated well with the literature reports [21,22]. The physical mixture of the drug and the various excipients had peaks correlating with the 2θ values of the drug albeit with lower intensity than the plain NM, which could be due to less concentration of API present in the physical mixture. The diffractogram of blank LPNs showed no peaks, suggesting the presence of all the ingredients in the amorphous form in the blank LPNs. The sharp, high-intensity peaks in the case of lyophilized NM-LPNs were primarily due to the presence of cryoprotectant (mannitol), which was confirmed by comparing with the pure mannitol pXRD data. In the lyophilized NM-LPNs diffractogram, the peaks that were specific to NM (non-interfering peaks of NM compared with other excipients) were missing, confirming the presence of NM in either amorphous or molecular form in the nanoparticles.

#### 3.5.3. Stability Studies

##### Particle Size Distribution and Morphology Evaluation Using SEM

**Figure 7:**
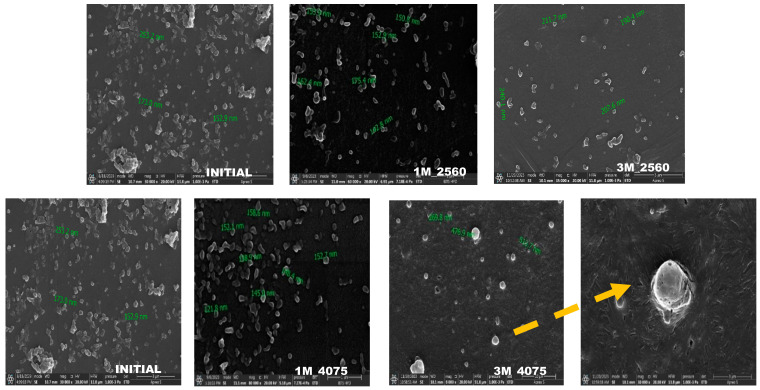
The SEM images of NM-LPNs samples at different time points during stability studies, INITIAL, 1M 2560 (1 month of storage at 25 ± 2 °C and 60 ± 5% RH), 3M 2560 (3 months of storage at 25 ± 2 °C and 60 ± 5% RH), 1M 4075 (1 month of storage at 40 ± 2 °C and 75 ± 5% RH), 3M 4075 (3 months of storage at 40 ± 2 °C and 75 ± 5% RH), and a porous LPN.

Scanning electron microscopy images were obtained to understand the changes in the PS and morphology of NM-LPNs during stability. The average PS of the NM-LPNs did not change significantly and remained below 200 nm over the 3-month long-term stability study at 25 ± 2 °C and 60 ± 5% RH. At accelerated stability conditions (40 ± 2 °C and 75 ± 5% RH), the PS and morphology were retained until 1 month of the study. However, in the 3rd month, the PS of NM-LPNs increased significantly compared to the initial PS. The particles appear to have become more porous at the end of 3 months of storage. Figure 7 shows SEM images for all the stability conditions.

PLGA is a heat- and moisture-sensitive polymer. With an increase in temperature and exposure to moisture, the degradation rate fastens. It undergoes degradation/erosion primarily due to hydrolysis and oxidation. However, the rate of degradation is dependent on the grade of PLGA. The porous and leaky structure of NM-LPNs after 1-month storage at accelerated conditions could be due to the hydrolysis and/or oxidation induced by both temperature and higher moisture content in the storage conditions. This can lead to drug leakage and, thereby, the loss of the drug from NM-LPNs when stored at higher temperature or humidity conditions.

##### % Assay Determination of NM-LPNs

**Figure 8:**
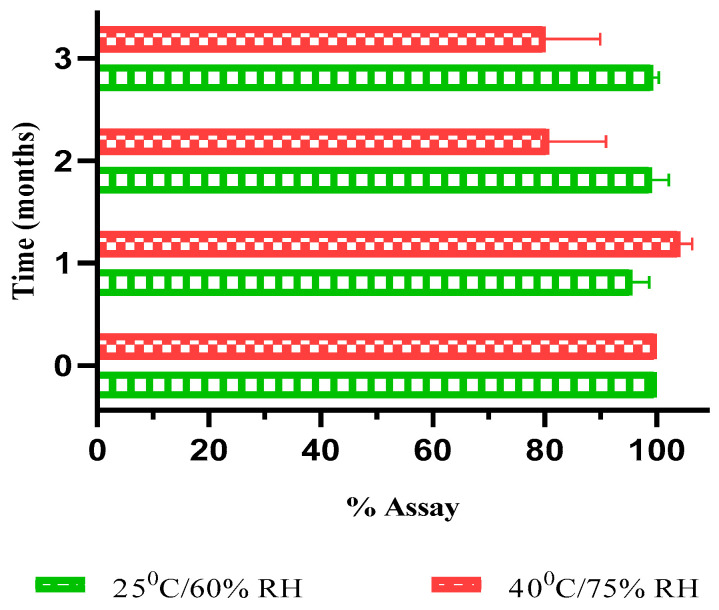
Drug content (% Assay) of NM-LPNs during 3 months of storage at 25 ± 2 °C and 60 ± 5% RH and 40 ± 2 °C and 75 ± 5% RH.

The results obtained from drug content (% Assay) analysis of NM-LPNs samples stored at 25 ± 2 °C and 60 ± 5% RH as well as 40 ± 2 °C and 75 ± 5%, during the 3-month stability study are presented in Figure 8. At 25 ± 2 °C and 60 ± 5% RH (CRT), the optimized NM-LPNs showed no difference in the drug content (99.38 ± 1.0%) over the 3-month stability study. At 40 ± 2 °C and 75 ± 5% RH (accelerated condition), the drug content of the optimized NM-LPNs did not change significantly till 1 month of storage. However, the NM-LPNs samples analyzed at the end of the 2nd month and 3rd month showed a 20% drop in the drug content compared to the same sample at time ‘t = 0’ (initial time point). These results correlate with the observations from SEM images of NM-LPN samples analyzed at the 3rd month of storage in accelerated conditions. The decrease in drug content after 1-month storage at accelerated conditions could be due to the porous and leaky nature of the LPNs due to the degradation of the PLGA (65:35) polymer.

Similar results were obtained through PS analysis using SEM, as described in the Section ‘Particle Size Distribution and Morphology Evaluation Using SEM’. In the SEM images, it was observed that after 1 month, at accelerated conditions, the NM-LPNs had become porous, which might have led to drug exposure to extreme temperature and humidity conditions. The degradation of the drug from 1 month onwards could be a result of structural modifications in NM-LPNs. These results suggest that the NM-LPNs should be stored at 25 ± 2 °C and 60 ± 5% RH to maintain the physicochemical stability of the formulation.

### 3.6. In Vitro Release Study for Plain NM and NM-LPNs

**Figure 9:**
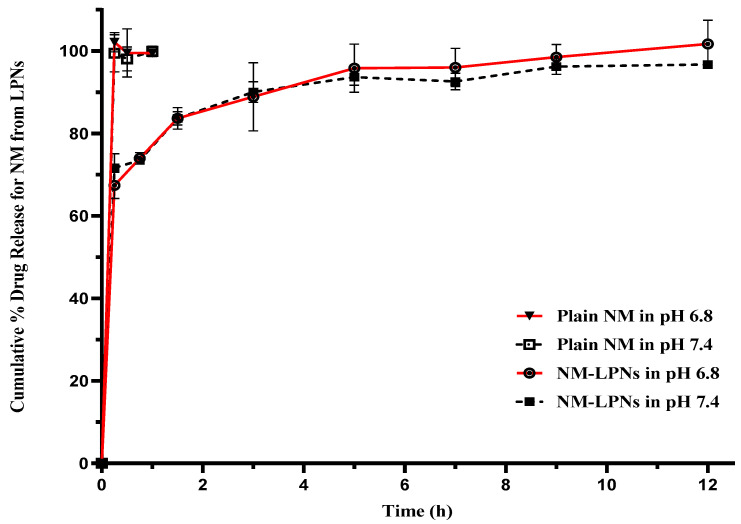
In vitro drug release profiles of plain NM and NM-LPNs in phosphate-buffer saline (pH 6.8 and 7.4) with 2% *w*/*v* of Gelucire 44/14. Note: The data are represented as mean ± SD of 3 replicates.

NM, being a lipophilic drug, does not have sufficient solubility in phosphate-buffer saline (pH 6.8 and 7.4) to maintain sink conditions during the in vitro drug release studies. Hence, solubility studies of NM were conducted in two different grades of Gelucire (Gelucire 59/14 and 44/14) to identify a suitable solubilizer to maintain the sink conditions in the in vitro drug release studies. NM showed the maximum solubility in 2% *w*/*v* solution of Gelucire 44/14. Therefore, the phosphate-buffer saline (pH 6.8 and 7.4) containing 2% *w*/*v* of Gelucire 44/14 was selected as the dissolution medium. The in vitro drug release profiles of plain NM and NM-LPNs at two different pH conditions are shown in Figure 9.

Plain NM dissolved completely (100%) within 5 min of the study in both pH conditions. In the case of NM-LPNs at pH 6.8, there was an initial burst release of 67.41 ± 3.19% within 15 min, which increased to 83.67 ± 1.63% at the end of 90 min. However, the drug release slowed beyond 90th min with 101.74 ± 5.75% drug release at the end of the 12th hour. Similarly, at pH 7.4, there was an initial burst release of 71.66 ± 3.43% within 15 min, which increased to 83.70 ± 2.62% at the end of 90 min. However, the drug release slowed beyond the 90th min with 96.75 ± 0.13% drug release at the end of the 12th hour. The observed release pattern could be due to the distribution of the drug loaded in the NM-LPNs. The initial burst release could be due to the presence of drug on the surface layers of the LPNs. This initial burst release can help in achieving plasma drug concentrations above the minimum effective concentration within a shorter period [17]. The sustained release of the drug up to 12 h can help in maintaining the plasma drug concentrations for a sufficiently longer duration.

The drug release data of NM-LPNs, when analyzed using zero-order, first-order, Higuchi, and Hixon Crowell kinetic models, resulted in correlation coefficient (R^2^) values of 0.792, 0.969, 0.924, and 0.766, respectively. The release of NM from NM-LPNs in a pH 6.8 dissolution medium is considered to follow first-order release kinetics based on the correlation coefficient values. Similar observations were obtained in the case of pH 7.4 dissolution media.

### 3.7. In Vivo Oral Pharmacokinetic Study

**Figure 10:**
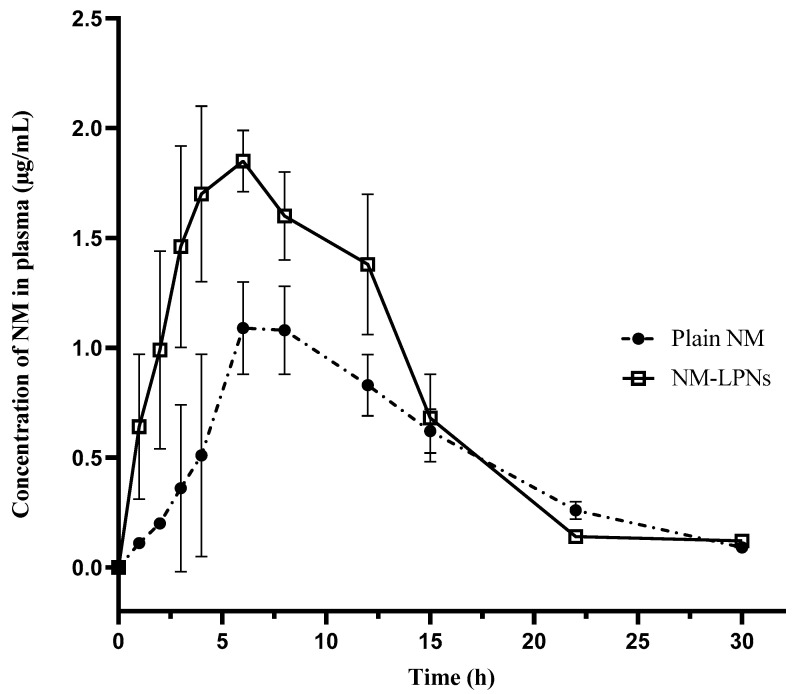
Plasma time course profile of plain NM and NM-LPNs in female Wistar rats at a dose of 10 mg/kg. Note: Each data point is presented as the mean ± SD for *n* = 3 rats.

In vivo oral pharmacokinetic studies in female Wistar rats showed significant differences in the plasma time course profiles of plain NM and NM-LPNs (Figure 10). The oral pharmacokinetic parameters of plain NM and NM-LPNs are mentioned in Table 5, while the raw data for individual rats obtained from pharmacokinetic studies of plain NM and NM-LPNs oral suspension are provided in Table S1. The mean C_max_ and the AUC_0–tlast_ values were significantly higher for NM-LPNs by 1.72 times (*p* < 0.01) and 1.58 times (*p* < 0.01), respectively, when compared to plain NM. However, no difference was observed in the mean residence time (MRT_last_) of plain NM and NM-LPNs. The higher C_max_ and AUC_0–tlast_ of NM-LPNs could be attributed to the direct uptake of nanoparticles either through paracellular or transcellular pathways, thereby reducing the chances of drug efflux and gut-wall metabolism of NM while absorbing through the gastrointestinal membranes.

Several different mechanisms were proposed by researchers for the direct uptake of nanoparticles through the gastrointestinal membrane, including clathrin and caveolin-mediated endocytosis, micropinocytosis, and lymphatic transport. The physicochemical properties of nanoparticles, including surface chemistry, PS, shape, and surface charge, can affect the mechanism(s) by which the nanoparticles are absorbed into the systemic circulation [23,24,25,26]. It would be interesting to elucidate the exact mechanism(s) involved in the uptake of NM-LPNs, resulting in the increased oral bioavailability of the drug compared to plain NM.

NM shows severe dose-dependent GI side effects, which lower the tolerance of therapy, and in extreme cases, the patient must discontinue the therapy. As it is a one-year chronic therapy, any duration less than that would disturb the therapeutic efficacy of the drug. While improving the bioavailability by 1.58 times, we anticipate a dose reduction of a minimum of 50%, which can help the patient sustain the therapy for a longer duration.

## 4. Conclusions

A hybrid lipid and polymer nanoparticulate system of NM using a novel combination of GDS and PLGA (65:35) was designed and optimized using DoE. X-ray diffractometry and thermal analysis data revealed that NM was loaded either in an amorphous or molecular state in the nanoparticles. The mean PS was 278.57 ± 21.16 nm, and the DL (%) was 25.77 ± 1.11% for the optimized NM-LPNs. The optimized NM-LPNs exhibited first-order drug release kinetics and sustained the drug release for 12 h in the in vitro dissolution studies in phosphate-buffer saline (pH 6.8). NM-LPNs could increase the C_max_ (1.72 times, *p* < 0.01) and AUC_0–tlast_ (1.58 times, *p* < 0.01) of the drug when administered through the oral route compared to the plain NM in the in vivo pharmacokinetic studies. With the increase in the relative oral bioavailability of NM-LPNs over plain NM, it is expected that the designed hybrid polymer and lipid nanoparticulate system of NM can reduce the oral dose of the drug. This can help reduce the dose-dependent side effects associated with the drug.

## Figures and Tables

**Table 1:** List of factors with their screening design (2^6−2^ Resolution IV) levels.

Factor	Factors Considered for Screening	Unit	Levels	
			Low (−1)	High (+1)
X_1_	Polymer amount	mg	2	6
X_2_	Lipid amount	mg	4	10
X_3_	Temperature	°C	6	20
X_4_	Tween 80 concentration in the organic phase	%	10	30
X_5_	Homogenization time	min	2	8
X_6_	Homogenization speed	rpm	7000	15,000

**Table 2:** Critical factors and their levels used in optimization design.

Factor	Name of the Factor	Unit	Levels		
			Low (−1)	Medium (0)	High (+1)
X_1_	Tween 80 concentration in the organic phase	%	1.33	2.665	4
X_2_	Lipid amount	mg	4	7	10
X_3_	Polymer amount	mg	10	20	30

**Table 3:** BBD matrix with the levels (in nominal scale) of each factor in various experimental runs and the corresponding responses, PS (nm) and DL (%), obtained for each run.

Run	Tween 80 Concentration in the Organic Phase (%)	Lipid Amount (mg)	Polymer Amount (mg)	PS (nm)	DL (%)
1	4	7	30	273.5	11.87
2	4	4	20	191.7	17.18
3	4	10	20	203.5	9.67
4	2.665	7	20	252.6	22.32
5	2.665	4	30	283.8	26.70
6	1.33	7	30	300.9	6.07
7	1.33	10	20	263.4	13.87
8	4	7	10	179	3.10
9	2.665	7	20	219.3	23.09
10	2.665	10	10	168	20.97
11	2.665	10	30	292	6.91
12	2.665	7	20	252.3	22.50
13	1.33	4	20	364.3	19.07
14	2.665	4	10	269.2	16.72
15	1.33	7	10	238.9	21.32

**Table 4:** ANOVA results for the adjusted best-fit models of the two responses (PS and DL (%)).

Source	Y_1__PS (nm)				Source	Y_2__DL (%)			
	SS	DF	F_cal_	*p* Value		SS	DF	F_cal_	*p* Value
Model	0.11	5	16.49	0.0003	Model	712.025	7	82.978	<0.0001
X_1_	0.039	1	30	0.0004	X_1_	42.798	1	34.913	0.0006
X_2_	0.012	1	9.11	0.0145	X_2_	99.742	1	81.366	<0.0001
X_3_	0.037	1	28.89	0.0004	X_3_	13.938	1	11.370	0.0119
X_1_X_2_	6.95 × 10^−3^	1	5.37	0.0458	X_1_X_3_	144.198	1	117.632	<0.0001
X_2_X_3_	0.012	1	9.09	0.0146	X_2_X_3_	144.578	1	117.942	<0.0001
Residual	0.012	9			X_1_^2^	205.959	1	168.014	<0.0001
Lack of Fit	9.17 × 10^−3^	7	1.05	0.5688	X_3_^2^	77.499	1	63.221	<0.0001
Pure Error	2.49 × 10^−3^	2			Residual	8.581	7		
Cor Total	0.12	14			Lack of Fit	8.260	5	10.311	0.0908
					Pure Error	0.320	2		
					Cor Total	720.606	14		

**Table 5:** Oral pharmacokinetic parameters of plain NM and NM-LPNs in female Wistar rats at 10 mg/kg dose of the drug.

Parameter	Plain NM ^@^	NM-LPNs ^@^
C_max_ (μg/mL)	1.11 ± 0.21	1.91 ± 0.23 **
T_max_ (h) ^b^	6.0	6.0
AUC_last_ (h·μg/mL)	15.16 ± 0.97	23.90 ± 1.93 **
MRT_last_ (h)	11.74 ± 0.84	9.38 ± 0.81 *
HL_Lambda_z (h)	5.31 ± 0.50	5.09 ± 0.28 *
F_rel_	-	1.58 **

^@^ The results are expressed as mean ± SD of n = 3 observations, and the plain NM data are part of our previous publication [18]. ^b^ The T_max_ values are represented as a median of *n* = 3 animals. * Statistically insignificant compared to the corresponding parameter of plain NM with *p* > 0.05. ** Statistically significant compared to the corresponding parameter of plain NM with *p* < 0.01.

## Data Availability

The original contributions presented in the study are included in the article/Supplementary Materials, further inquiries can be directed to the corresponding author.

## Supplementary Materials

The following supporting information can be downloaded at: https://www.mdpi.com/article/10.3390/pharmaceutics17020221/s1, Table S1: Raw data for individual rats obtained from pharmacokinetic studies of plain NM and NM-LPNs oral suspension.
