# Supplementary material for: Oral Administration of Neratinib Maleate-Loaded Lipid–Polymer Hybrid Nanoparticles: Optimization, Physical Characterization, and In Vivo Evaluation
Source: Pharmaceutics. 2025 Feb 8;17(2):221. doi: 10.3390/pharmaceutics17020221 (PMC11858839; doi:10.3390/pharmaceutics17020221)
Supplement: Supplementary file 1 [file pharmaceutics-17-00221-s001.zip › pharmaceutics-3438022-supplementary.pdf]

SUPPLEMENTARY DATA

Table S1 Raw data for individual rats obtained from pharmacokinetic studies of plain NM and NM-LPNs oral suspension

Time (h)	Plain NM					NM-LPNs				
	Rat 1	Rat 2	Rat 3	AVG	SD	Rat 1	Rat 2	Rat 3	AVG	SD
1	0.00	0.00	0.34	0.11	0	1.01	0.49	0.41	0.64	0.33
2	0.00	0.00	0.59	0.20	0	1.50	0.67	0.79	0.99	0.45
3	0.33	0.54	0.76	0.54	0.21	1.99	1.16	1.22	1.46	0.46
4	0.68	0.77	0.86	0.77	0.09	2.15	1.58	1.39	1.70	0.40
6	1.00	1.33	0.93	1.09	0.21	1.96	1.89	1.69	1.85	0.14
8	1.05	1.29	0.90	1.08	0.20	1.50	1.83	1.46	1.60	0.20
12	0.82	0.97	0.70	0.83	0.14	1.06	1.69	1.39	1.38	0.32
15	0.60	0.72	0.53	0.62	0.10	0.48	0.89	0.69	0.68	0.20
22	0.24	0.31	0.25	0.26	0.04	0.15	0.14	0.13	0.14	0.01
30	0.07	0.10	0.09	0.09	0.02	0.11	0.12	0.11	0.12	0.01

AVG - Average; SD - Standard deviation (calculated for n=3 animals in each group).
